# Fentanyl Overdose Causes Prolonged Cardiopulmonary Dysregulation in Male SKH1 Mice

**DOI:** 10.3390/ph17070941

**Published:** 2024-07-14

**Authors:** Mackenzie Newman, Heather Connery, Swapna Kannan, Aarti Gautam, Rasha Hammamieh, Nabarun Chakraborty, Jonathan Boyd

**Affiliations:** 1 Department of Orthopaedic Surgery, Virginia Commonwealth University School of Medicine, Richmond, VA 23298, USA; mackenzie.newman@vcuhealth.org; 2 Department of Physiology, Pharmacology and Toxicology, Robert C. Byrd Health Sciences Center, West Virginia University, Morgantown, WV 26506, USA; 3 Walter Reed Army Institute of Research, Silver Spring, MD 20907, USA

**Keywords:** fentanyl, overdose, overdose survival, immunosuppression, LD50, cardiotoxicity, cardiopulmonary system

## Abstract

Fentanyl overdose is a survivable condition that commonly resolves without chronic overt changes in phenotype. While the acute physiological effects of fentanyl overdose, such as opioid-induced respiratory depression (OIRD) and Wooden Chest Syndrome, represent immediate risks of lethality, little is known about longer-term systemic or organ-level impacts for survivors. In this study, we investigated the effects of a single, bolus fentanyl overdose on components of the cardiopulmonary system up to one week post. SKH1 mice were administered subcutaneous fentanyl at the highest non-lethal dose (62 mg/kg), LD10 (110 mg/kg), or LD50 (135 mg/kg), before euthanasia at 40 min, 6 h, 24 h, or 7 d post-exposure. The cerebral cortex, heart, lungs, and plasma were assayed using an immune monitoring 48-plex panel. The results showed significantly dysregulated cytokine, chemokine, and growth factor concentrations compared to time-matched controls, principally in hearts, then lungs and plasma to a lesser extent, for the length of the study, with the cortex largely unaffected. Major significant analytes contributing to variance included eotaxin-1, IL-33, and betacellulin, which were generally downregulated across time. The results of this study suggest that cardiopulmonary toxicity may persist from a single fentanyl overdose and have wide implications for the endurance of the expanding population of survivors.

## 1. Introduction

Fentanyl is a deadly narcotic drug responsible for more deaths than all other opioids combined [1], with over 100,000 deaths in 2023 in the United States alone. Overdose is common due to the high potency of fentanyl and its derivatives, where quantities as low as 50 µg have been reported to cause severe respiratory depression requiring emergency medical attention [2]. Even in clinical settings, titration is critical, because low anesthetic doses may produce Wooden Chest Syndrome, which typically requires intubation and cholinergics, in addition to an opioid antagonist such as naloxone [3]. While fentanyl overdose deaths dominate media attention, there are more survivors each year than mortalities. Rates of survival from fentanyl overdose have not been specifically characterized, but it is estimated that there are five survivors for each opioid overdose death [4].

Most fentanyl studies focus on neural, behavioral, and physiological impacts around the acute period of intoxication, using relatively low doses (e.g., less than 1 mg/kg [5,6,7] compared to an LD50 up to 135 mg/kg in mice [8]). Following fentanyl administration, opioid-induced respiratory depression (OIRD) occurs due to μ opioid receptor (MOR) agonism in the medulla oblongata (particularly in the pre-Bötzinger complex), decreasing breathing and leading to systemic hypoxia [9]. In overdose, individuals may become fully apneic [10], potentially producing organ or tissue damage within minutes [11], yet nonfatal OIRD typically resolves without intervention. In humans, OIRD generally occurs for up to 8 h [12], while in mice, it has been shown to abate within 40 min from fentanyl overdose [8] and persist up to 8 h after morphine overdose [13].

Following fentanyl overdose, the resolution of OIRD is implicit for survival, but the longer-term organ-level impacts have been poorly characterized. Outside of neurological impacts such as depression [14] and impaired cognition [15], opioid use has been associated with the risk of many cardiovascular diseases [16], yet in acute settings, opioids have been referred to as cardioprotective [17]. Non-cardiogenic pulmonary edema may occur acutely with opioids [18,19], and was reported in 96% of fentanyl overdose fatalities in a small study, in contrast to 54% with cerebral edema in the same study [20]. Evidence of long-term changes in airway resistance is mixed [19,21], but a risk of pneumonia [21] and tuberculosis [22] are frequently concomitant with opioid abuse [19].

Broadly, opioids are considered immunosuppressive [23], decreasing natural killer (NK) cell cytotoxicity and macrophage function in rodents in vivo, but cytokine concentrations (e.g., TNF-α [24,25,26,27], IL-1β [27,28], and IL-6 [29,30]) from primary human in vitro samples [25] have been inconclusive (both increasing, decreasing, or having no significant effect) [30,31]. While a few studies have shown immunosuppression from fentanyl in mice [25], the temporal progression of these changes has not been shown previously. Fentanyl presents unique considerations compared to conventional opioids, with implications for immune activity, such as an augmented binding mode [32], off-site binding [33], and high lipophilicity that could result in a delayed release and impacts [34].

In a previous study, we demonstrated the lethality of fentanyl in SKH1 mice and its immediate cardiopulmonary effects [8]. Using our validated and reproducible dosing methodology from that study, we now present an investigation into the additional effects of acute fentanyl overdose on the cerebral cortex, heart, lungs, and plasma, using a broad immunomodulatory surveillance panel, for up to one week post-administration. Given expanding rates of overdose and therefore overdose survivors, the results of this study have wide implications for future public health.

## 2. Results

In these studies, SKH1 mice were exposed to HNLD, LD10, and LD50 concentrations of fentanyl following previously determined toxicity values [8]. Subject responses were in line with fentanyl overdose, presenting full apnea within 30 s of drug administration, then slowly resolving with visible shallow breathing up to one minute later. Many subjects exhibited ocular blanching within an hour, presumably due to hypoxia, which recovered by the following day. By six hours post-administration, most animals had generally returned to normal behavior. These doses were determined for a 48-hour survival threshold, and while non-survivors typically died within one hour of fentanyl administration, there is still a risk that non-survivors were present at early time points (see “Discussion” for further detail). Tissue and plasma samples were taken at 40 min, 6 h, 24 h, and 7 d following fentanyl exposure and analyzed using a 48-analyte multiplex, surveying various cytokines, chemokines, and growth factors. The complete results are shown in Figure S1, and briefly described below. In comparison with controls that received only Ringer’s solution, the results generally followed a dose-dependent response. Recovery to baseline occurred at 7 d post in most samples, excluding the heart, where lower fentanyl doses (HNLD, LD10) had the most prominent, significant long-term effects.

**Figure 1:**
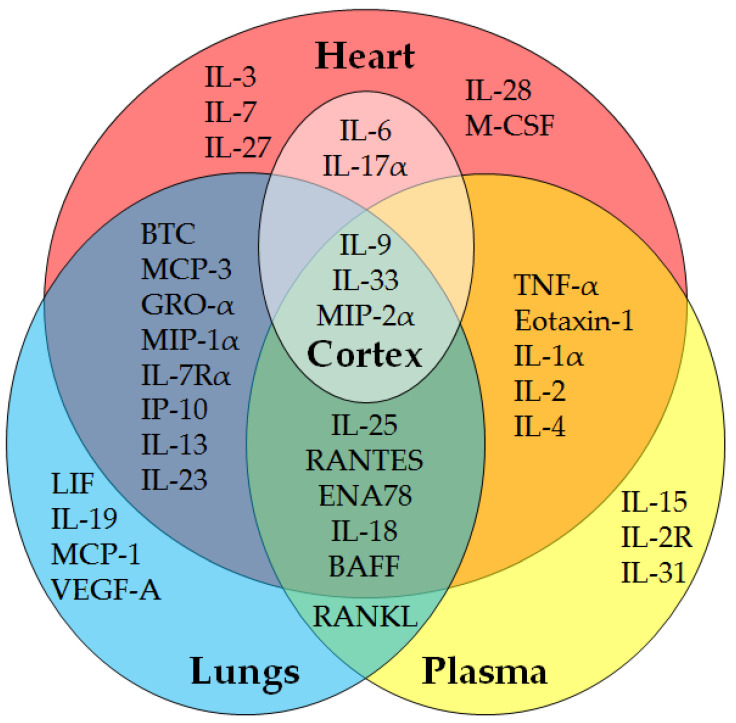
Fentanyl overdose analytes with significance at any time point x dose via 48 plex. Fentanyl overdose (62–135 mg/kg, s.c. bolus) samples were collected along with vehicle (Ringer’s solution) controls post-drug administration (40 min–7 d) and processed using ProcartaPlex Mouse Immune Monitoring 48-Plex Panels. After removing outliers, significance (*p* < 0.05) versus time-matched controls was determined via two-way ANOVA or mixed-effects model (*n* = 3–4 per time point x dose; N = 64).

A summary Venn diagram (Figure 1) of protein changes across organs demonstrates a wide range of impacts from fentanyl overdose with many cardiopulmonary effects. Figure 1 displays analytes that were significantly different (*p* < 0.05, increased or decreased) from control, regardless of dose or time point. The cortex revealed five analytes with significance; three were shared with the heart, lungs, and plasma (IL-9, IL-33, and MIP-2α) and two were shared with the heart exclusively (IL-6, IL-17A). In contrast, plasma presented three unique proteins (IL-2R, IL-15, and IL-33) out of 17 total, lungs had four unique analytes (LIF, IL-19, MCP-1, and VEGF-A) out of 21 total, and the heart had the most, with five unique analytes (IL-3, IL-7, IL-27, IL-28, and M-CSF) out of 28 total. Heart, lungs, and plasma all presented significant changes in IL-25, RANTES, ENA78, IL-18, or BAFF. While the plasma and lungs only had one common analyte, RANKL, the plasma and heart shared five proteins (eotaxin-1, IL-1α, IL-2, IL-4, and TNF-α), and the heart and lungs shared eight (IL-13, IL-23, IP-10, IL-7Rα, GRO-α, BTC, MIP-1α, and MCP-3).

**Figure 2:**
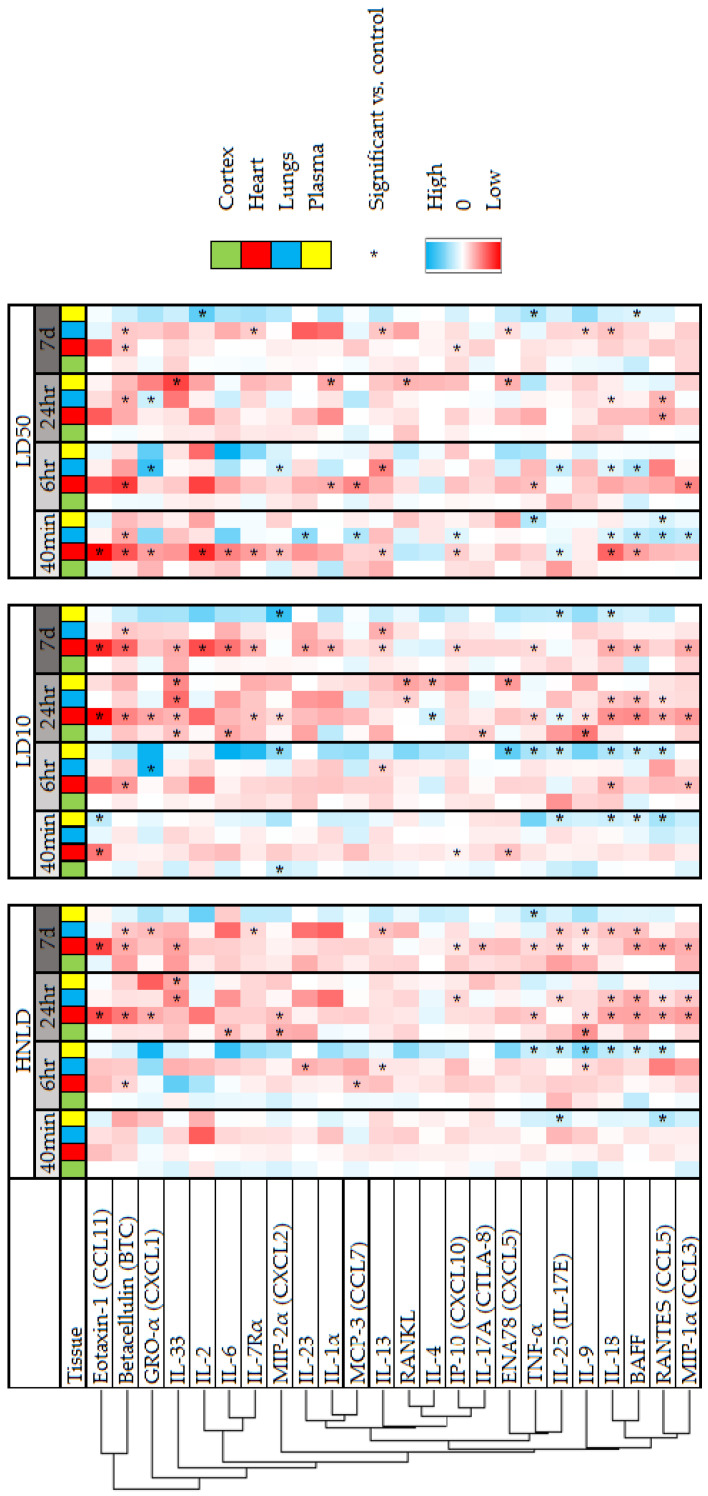
Distribution of shared cardiopulmonary fentanyl overdose analytes via non-supervised hierarchical clustering. Fentanyl overdose (62, 110, or 135 mg/kg; s.c. bolus) samples were collected along with vehicle (Ringer’s solution) controls at 40 min, 6 hr, 24 hr, or 7 d post-drug administration and processed using ProcartaPlex Mouse Immune Monitoring 48-Plex Panels. After removing outliers, significance versus time-matched controls (indicated by *; *p* < 0.05) was determined via two-way ANOVA or mixed-effects model (*n* = 3–4 per time point x dose; N = 64). Analytes with significance in only one organ or plasma were removed.

Stratifying the data by dose and time, Figure 2 shows a heatmap summary of normalized log_2_-transformed concentration values across all organs and plasma for analytes shared with significance in at least two organs or plasma, derived from concentration data in Figure S1. Broadly, HNLD and LD10 doses appear to elicit similar responses, distinct from the LD50 dose: at early time points (40 min, 6 hr), HNLD and LD10 caused few significant changes across all samples (8.9 ± 2.3% of all analytes), in comparison to widespread significant decreases in heart and lung tissue at later time points (24 hr, 7 d; 22.7 ± 1.9% of all analytes). In contrast, LD50-treated subjects had a greater response earlier when compared to later time points (17.2 ± 5.8% vs. 6.3 ± 3.1% of all analytes). Unsupervised hierarchical clustering reveal a segregation of cardiac-specific responses (e.g., eotaxin-1, betacellulin (BTC), and GRO-α) from significant analytes found across the heart, lungs, and plasma (e.g., RANTES, BAFF, and IL-18).

The coherence between the HNLD and LD10 doses compared to LD50 is further corroborated by Figure S2, showing PCA (Principal Component Analysis) plots of individual organs and plasma. The two-dimensional PCA of the heart samples (Figure S2A) explained nearly 53% of the total variance, and samples with the two lower doses clustered together, showing a higher coherence between them than LD50. In comparison, a time-specific and largely dose-independent clustering pattern emerged in both plasma (Figure S2B) and lungs (Figure S2C) samples. Nearly 58% and 64% of the total variance were explained by the two-dimensional PCA plots of the lungs and plasma, respectively. The cortex showed a minimum variance among all the tissue types (Figure S2D), where its two-dimensional PCA described nearly 98% of the total variance. The PCA of all samples, displayed in Figure S3, shows the divergence of heart and plasma tissues, with a large overlap with lungs and cortex tissue.

Using a supervised learning approach to determine the PCA loading (Figure S4), we curated the analytes with the largest contribution to variance in the study. Here, PC1 and PC2 explained 24.98% and 11.95% of the variance profile caused by all cofactors (tissue, dose, and time). The protein changes with the greatest contribution to PCs were filtered into Table 1. Setting the cut-off at |0.2|, proteins with the greatest magnitude on PC1 include eotaxin-1, BTC, and IL-7, while IL-33, RANTES, and BAFF were involved in variance associated with PC2.

**Figure 3:**
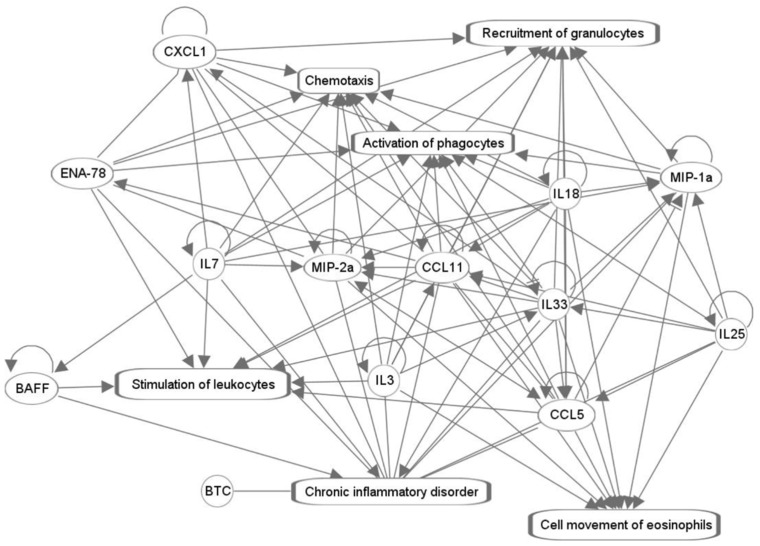
Biofunctions linked to analytes with maximum contribution to variance. Proteins with the greatest magnitude of response from Table 1 were used to generate a network of potential biofunctions associated with fentanyl overdose. The nodes and edges represent the analytes and their relationships, accordingly. The solid lines represent their association, and pointed arrowheads denote the activating relationship between the two nodes Major biofunctions included chronic inflammatory disorder (13 nodes), activation of phagocytes (10 nodes), chemotaxis (9 nodes), recruitment of granulocytes (9 nodes), stimulation of leukocytes (8 nodes), and cell movement of eosinophils (8 nodes).

The analytes with the greatest contributions to PC1 and PC2 loading values were used to generate the network of biofunctions presented in Figure 3. Due to a low sample number (n = 3–4 per tissue x time point x dose group), the pathway was generated regardless of directionality (i.e., activation or inhibition based on the increase or decrease in protein concentration). The central functions linked to these cytokines, chemokines, and growth factors, ranked by number of nodes, include chronic inflammatory disorder (13 nodes: BTC, ENA78, BAFF, IL-7, CXCL1, MIP-2α, IL-3, CCL11 (eotaxin-1), IL-18, IL-33, CCL5) RANTES), IL-25, MIP-1α), activation of phagocytes (10 nodes: MIP-1α, IL-25, CCL5, IL-33, IL-18, CCL11 (eotaxin-1), IL-3, IL-7, CXCL1, ENA78), granulocyte recruitment (9 nodes: GRO-α (CXCL1), ENA78, IL-7, MIP-2α, eotaxin-1 (CCL11), IL-18, IL-25, IL-33, MIP-1α), chemotaxis (9 nodes: ENA78, GRO-α (CXCL1), IL-7, MIP-2α, IL-3, CCL11, IL-33, MIP-1α, IL-18), eosinophil movement (8 nodes: eotaxin-1 (CCL11), IL-3, IL-18, IL-25, IL-33, MIP-1α, and MIP-2α, and RANTES (CCL5)), and stimulation of leukocytes (8 nodes: BAFF, ENA78, IL-7, CCL11. IL-18, IL-33, IL-3, and CCL5).

## 3. Discussion

Overdose is a complex phenomenon that is poorly defined or understood. In these studies, we used three doses of fentanyl—62, 110, and 135 mg/kg, representative of HNLD, LD10, and LD50, respectively, for SKH1 mice—which are exceptionally high compared to typical studies [35,36,37]. These concentrations cause dose-independent effects on breathing, suggesting the saturation of the respiratory pathways and the potential for spill-over into off-target receptors [8]. Fentanyl has been described as having substantial affinities for monoamine receptors, especially α_1_-adrenergic receptors (α_1B_ ≥ α_1A_ > α_1D_) [33], which have been implicated in vascular comorbidities unique to fentanyl among all opioids [38]. α_1_ receptors are present throughout the brain, heart, and lung tissues, and are enriched in vascular smooth muscle. The activities of other receptors with a non-negligible affinity for fentanyl, such as M_3_ [39], 5-HT_1a_ [40], and TLR4 [41], have not been clarified. Hypoxia resulting from OIRD can also confound receptor activity, as many of these receptors, along with MOR itself, can impact and be impacted by downstream oxygen sensors such as HIF-1α [42,43,44].

Upon high-dose fentanyl administration, a decrease in blood oxygen is typically preceded by bradycardia [8]. The early initiation of this process is prognostic for long-term survival, yet in our experience with overdose in SKH1 mice, some non-surviving subjects live for more than 40 min (i.e., average time of death was 44 min) [8]. The dataset herein contains “survivor bias” (i.e., all subjects survived), as there is always the risk that some subjects were on a trajectory to death, especially at earlier time points and the LD50 dose, where, by definition, 50% were terminal. While the results from the PCA plots (Figures S2 and S3) revealed a grouping of HNLD and LD10 samples in the heart, lungs, and plasma at the 40 min and 6 h time points, hierarchical clustering revealed a group of proteins with significant changes at 24 h in the HNLD and LD10 doses that were not present with LD50. At the 40 min time point, there were relatively few significantly different analytes across all samples at the HNLD and LD10 doses, yet LD50 generally lead to significant decreases across heart analytes (e.g., IL-2, IL-6, eotaxin-1, and BTC) and significant increases in plasma and lung analytes (e.g., IL-23, BAFF, and IL-18). While the roles of these proteins in the context of mortality is outside the scope of this study, they may be critical mediators of the acute lethal response to fentanyl. For example, elevated levels of RANTES, TNF-α, and MCP-3 have been associated with a lethal cytokine storm in COVID-19 patients [45].

Opioids have been discussed to be broadly immunosuppressive [25,31,46], yet because small structural differences between opioids mediate differential immune responses [47], generalizations about immunomodulation by MOR agonists should be observed with circumspection. For example, while fentanyl and other opioids have been shown to decrease natural killer (NK) cell cytotoxicity in humans and rodents [46], fentanyl has been shown to not have a significant effect on mast cells from donor heart, lung, and skin isolates [48], which is often observed with other opioids [49,50].

Tissue-specific context is critical in the timecourse of the immunological response to fentanyl. For example, the rapid onset of fentanyl is attributed to its lipophilicity [32], yet active transport at the blood–brain barrier by ABCB1 [51] and OATP1A2 [52] has also been suggested to play a role in uptake and retention. Fentanyl may destabilize the blood–brain barrier as well [37], though its effects on maintaining or restricting cerebral blood flow have been inconclusive [53,54,55]. In this study, the cerebral cortex was assayed due to its contributions to voluntary respiration and as a spatially distal component of the cardiopulmonary system. Few analytes were detected above the lower limit of detection in the cortex, and those that were significantly different than controls were sparse, suggesting subtle effect in this region. IL-6, a widely reported pro-inflammatory factor [56], was significantly reduced after 24 h at the HNLD and LD10 doses; in contrast, a recent study using a low-dose chronic administration mouse model showed increased IL-1β, IL-6, and TNF-α transcript abundances in the cortex [54]. The effects of acute overdose versus chronic low-dose fentanyl have not been explored, but this differential response should elicit further research.

Circulating fentanyl has been shown to be largely sequestered into human lungs and partially expired [57], with the remainder principally metabolized to MOR-inactive norfentanyl [58]. Retention in the lungs may serve as a slow-release reservoir leading directly to the heart and into the circulation: at high doses, a second peak of activity has been observed, due to slow tissue release kinetics [34]. In humans, the elimination half-life is described somewhere between 3 and 10 h [59,60], yet elimination data are sparse in mice. Opioids have been described as cardioprotective [17], rendering fentanyl and higher-potency derivatives ideal replacements for traditional surgical anesthetics. The heart and lungs shared the greatest number of analytes responding differently to controls (8), with common reductions at HNLD and LD10 doses but not LD50. Of these proteins, only 3 (BTC, MIP-1α, and GRO-α) are among the 13 with the greatest effect on component loading (Table 1). BTC, an epidermal growth factor receptor ligand, was significantly decreased for 3 of the 4 time points measured in this study for each dose in the heart, suggesting a persistent effect that requires clarification given the role of BTC in vascular smooth muscle regulation and proliferation [61,62]. The reduced expression of BTC has not been explored in the heart and lungs, but genetic overexpression leads to increased lung and heart weight and hypertrophy [63]. BTC knockout mice have been investigated in cancer [64] and have “no overt defects” [65], yet decreased endogenous BTC has not been explored in the heart and lungs.

A coordinated interplay between major components of the cardiopulmonary system is critical for survival after toxic exposure, with several cross-impacted organ axes, as shown in Figure 1. However, common significant analytes between the heart, lungs, and plasma were largely incoherent across time and dose. Most of these (BAFF, RANTES, IL-18, and ENA78) are contributors to major component loading, at low magnitude. For all but ENA78, whose significance is sparsely distributed across samples, heart and lung concentrations were both reduced at 24 h at low doses, with incomplete resolution at 7 d. Plasma levels of these pro-inflammatory factors were significantly elevated, in contrast, at the initial time points (40 min, 6 h) and low doses but were largely attenuated by 24 h. IL-18 receptor activation by IL-18 has been shown to stimulate RANTES production [66], but discordant lung and heart concentrations suggest an insensitivity that requires validation. Further, BAFF, a B-cell activating factor that promotes antibody production and can be induced by IL-18, is significant at the same time points as IL-18 across doses. LD50 exposures produced a significantly increased BAFF and IL-18 in lungs at 40 min and 6 h, while they were only significantly increased in plasma at lower doses. These proteins go against the overall trend of reduced concentrations of all analytes in the lungs across the study, suggesting a secondary reaction to fentanyl that may be related to survival. Elevated lung concentrations of IL-18 and BAFF have been associated with chronic obstructive pulmonary disorder (COPD) [67,68], but the long-term nature of COPD progression is vastly different from the acute overdose response demonstrated herein. Nonetheless, tidal volume, a critical respiratory parameter implicated in expiratory function, has been shown to be decreased in COPD; in our prior study using the SKH1 model of fentanyl overdose, we demonstrated that a significantly decreased tidal volume was one factor implicated for survival [8]. Clarification of the inverted responses of IL-18 and BAFF may be critical in understanding the first 24 h of inflammation induced by fentanyl; further, these may also be related to longer-term physiological changes associated with COPD, but this requires additional research.

IL-33 was the sole analyte that was significantly decreased systemically across the cortex, heart, lungs, and plasma simultaneously, occurring in all LD10 samples at 24 hrs post-fentanyl exposure and other various time points/doses. As the highest-magnitude component of principal component 2, its paracrine function in endothelial barrier maintenance and activation may be relevant in overdose immunophysiology. IL-33, a pro-Th2 chemotactic that can attenuate TLR4 activity [69], is pro-inflammatory in the heart and lungs at elevated concentrations [70]. The activity of cognate receptor ST2 has been associated with cardiac mechanotransduction [71] and various cardiovascular diseases such as left ventricular hypertrophy [72] and heart failure [73]. Paradoxically, the genetic ablation of IL-33 leads to asthma and cardiac hypertrophy [74]; at HNLD and LD10 doses in this study, cardiac IL-33 was still significantly decreased at day 7, suggesting a long-term impact warranting further elucidation.

Eotaxin-1 (CCL11), the greatest-magnitude contributor to PC1, was significantly decreased in the heart primarily at 24 h and 7 d after fentanyl administration across doses. It is requisite for basal heart eosinophil maintenance in a Th2-dependent manner [75]. Derived from cardiac fibroblasts and macrophages [76], eotaxin-1 has been shown to decrease coronary endothelial junction components claudin-1, occludin, and zonula occludens-1, promoting monolayer permeability in vitro [77]. The reduced eotaxin-1 observed across heart samples in the present study may, therefore, contribute to decreased cardiac vascular compliance, but further research is necessary. One study has shown that CCL11 knockout mice exhibited no gross cardiac histological changes, but it did not report functional outcomes [78]. Eosinopenia resulting from a single bolus of a small molecule (e.g., catecholamines, cortisol) is typically transient [79]; chronic cardiac eosinopenia, suggested in this study by low eotaxin-1, may be the result of tissue damage. In humans, post-myocardial infarction blood eosinopenia has been associated with degree of inflammation and infarct severity [80,81], but this study did not have follow-up measurements after initial clinic visits to explore the long-term implications. Neither eosinopenia nor eosinophilia appear to be associated with fentanyl or the other common opioid literature beyond a case report concerning a transdermal fentanyl patch [82], and our findings support future research efforts in this area.

Cardiotoxicity following fentanyl administration persisted for the length of this study at HNLD and LD10 doses across many analytes. No previous studies have directly addressed chronic cardiac impacts from single, acute fentanyl overdose, although individuals with chronic opioid use disorder are at an increased risk of developing atherosclerosis and microvascular dysfunction [16]. They also exhibit significantly decreased high-frequency responses via heart rate variability analysis [16]. This finding, implying a reduced parasympathetic tone correlated to tachycardia and atrial fibrillation [83], may be due to tolerance and therefore not be present in our naïve overdose model; heart rate variability has not been reported in overdose survivors, despite the widespread contemporary availability of wristband ECG monitoring devices capable of heart rate variability assessment. Surrogate markers of cardiac changes may be vital for detecting the long-term effects of fentanyl overdose in humans, given the inverse relationship between plasma and tissue analyte concentrations and a limited access to biopsy tissue.

The biofunction analysis of proteins from Table 1 yielded the sequestration of two analytes by activity in Figure 3: BTC was linked solely to chronic inflammatory disorder and BAFF was involved with that and the stimulation of leukocytes. All other proteins had an overlap in multiple functions, including the movement of eosinophils, stimulation of leukocytes, activation of phagocytes, recruitment of granulocytes, and chemotaxis. All the remaining analytes that have not yet been discussed (MIP-2α, RANTES, IL-25, GRO-α MIP-1α, IL-3, and IL-7) were significant in the heart, with IL-3 and IL-7, which promote the maturation of hematopoietic stem cells into lymphoid progenitor cells [84,85], insignificant in other samples. MIP-2α, a pro-angiogenic marker [86,87], appeared in all three tissues and plasma. RANTES, a T-cell and monocyte-derived pro-inflammatory factor [88], and IL-25, a pro-Th2 cytokine [89], were significant in the heart, lungs, and plasma; GRO-α, a marker of chronic inflammation [90], and MIP-1α, an inflammatory secreted primarily from mature hematopoietic cells [91], were shared by the heart and lungs. The roles of these analytes in fentanyl overdose have not been previously characterized and require further validation.

### Limitations

The results of this work should be taken with caution, like all basic pharmacology studies. The term “overdose” is often poorly defined: it involves the administration of an excessive amount of an agent, producing a toxic effect, which was a potential for lethality in the present work. In both humans and mice, fentanyl is unique for its potentially idiopathic, dose-independent induction of fatal Wooden Chest Syndrome; otherwise, a lower bound for a lethal dose has been estimated at around 27 μg/kg in humans [92]. Mice tolerate many opioids several orders of magnitude more greatly (>1000x), with a 50% survival up to 135 mg/kg with fentanyl [8]. Beyond species differences in doses, changes in the MOR sequence and function are confounded by widespread human polymorphisms that impact signaling. The MOR is uncharacterized in SKH1 mice, the hairless yet immunocompetent strain used in this study. Sample size is also a limitation, as four subjects per time point x dose is the minimum number that is sufficient for statistics, given outliers and sample loss due to equipment malfunction. Survivor bias, described in further detail below, is also a confounder, with the greatest impact at later time points. Finally, the data presented herein are only focused on protein responses, and an integrative approach with transcription, metabolic, genetic, and epigenetic data would help us comprehend a more complete understanding of the impact of fentanyl.

## 4. Materials and Methods

All procedures were evaluated and approved by the Institutional Animal Care and Use Committee at West Virginia University (WVU IACUC) and United States Army Medical Research and Development Command Animal Care and Use Review Office (USAMRMC ACURO).

### 4.1. Animals

Male SKH1 Elite mice (strain code 477, Charles River, Wilmington, MA, USA; n = 64), aged 9–13 weeks (30.8 ± 3.07 g), were used in these experiments, and were group-housed (five per cage) in a vivarium at 40%–60% humidity and 20–24 °C with a 12 h light/dark cycle. All experiments were conducted during the light portion of the cycle in a surgical suite. After drug treatment, the subjects were single-housed. Animals had ad libitum access to Envigo Teklad 2918 chow and water. An acclimation period of at least 3 d was provided for all animals. Individual subjects were randomly selected during experiments. SKH1 mice were chosen for this study to follow previous work establishing fentanyl lethality in this strain. Animal numbers were kept minimal (n=4 per group) to satisfy a power analysis based on previous data.

### 4.2. Drug Treatment

The procedures were identical to a previous study [8]. Briefly, fentanyl citrate (Spectrum Chemical, Gardena, CA, USA; #F1147, lot 1JC0116, 99.2% purity) was dissolved in Ringer’s solution (Fisher Scientific, Waltham, MA, USA; S25513, lot 0GI20080611A) at 25 mg/mL and diluted to volumes of 80–200 µL depending on dose and body weight. Subjects received either fentanyl at 62, 110, or 135 mg/kg (highest non-lethal dose [HNLD], LD10, and LD50, respectively) or 200 µL Ringer’s solution (to maximize the effect from volume). After a brief induction of anesthesia with 1.5–2% isoflurane, subcutaneous injections were performed on the back of the neck, and anesthesia was ceased. All fentanyl administrations were performed with an assistant holding naloxone in case of emergency. All animals were monitored for 30 min following drug administration, then at least once every thirty minutes for six hours. During observation, cages were half-placed on a heating pad. Animals were humanely euthanized at 40 min, 6 h, 24 h, or 7 d post-administration, with organs flash-frozen in liquid nitrogen for future analysis.

### 4.3. Biomarker Assays

ProcartaPlex™ Mouse Immune Monitoring 48-Plex (EPX480-20834-901, lot 285653-001, ThermoFisher Scientific, Waltham, MA, USA) kits were employed, using the cerebral cortex, heart, lungs, or plasma. Tissues were cryoground, before being sonicated (4 sets of 2 s pulses, with 1 s pause between at 35% amplitude) in ProcartaPlex lysis buffer (EPX-99999-001; ThermoFisher Scientific, Waltham, MA), then normalized to 1.2 mg/mL using Bradford’s assay. Plasma was prepared using potassium EDTA collection tubes according to the manufacturer’s instructions (Microvette CB 300 K2E, Sarstedt AG + Co. KG, Nümbrecht, Germany)). For all kits, the manufacturer’s instructions were followed during assay, using a Biotek 405 ts magnetic plate washer. Briefly, beads were added to a black clear-bottom 96-well plate and incubated with samples and a dilution series of standards overnight. After washing, the beads were then incubated with primary antibodies, washed, incubated with secondary antibodies, washed, then introduced to a final reading solution. Plates were read using a Luminex xMAP INTELLIFLEX according to the manufacturer’s printed instructions, except for the DD gate, which was set to 7000—17,000, per technical support from ThermoFisher. Revision A.0 (30) of the product manual incorrectly states the DD Gate as 4000—13,000. All samples, standards, and blanks were assayed in duplicate.

### 4.4. Data Analysis

Raw Luminex data were trimmed to median fluorescence intensity (MFI), averaged, and background-subtracted before being fit to standard concentration curves using 5-parameter logistic regression in JMP (version 17.2.0) with all r^2^ > 0.97. After replacing analytes detected below the lowest standard with the lower limit of detection and removing outliers with the non-iterative Grubbs’ test, the analytes were compared to controls using the 2-way ANOVA or mixed-effects models. Significance was set to *p* < 0.05.

Analyte expression values were log-transformed and Principal Component Analyses (PCAs) were performed for each organ separately and on the combined dataset using the open source Tidyverse R package [93], and visualized using the ggplot2 package. PCAtools (version 2.16.0, accessed on 1 March 2024; [94],) was used to generate the loading scores corresponding to the individual analyte’s contribution to the top five PCs. We used R scripts to generate the hierarchical clustering based on the Euclidean distance algorithm. A functional network analysis was conducted using Ingenuity Pathway Analysis (QIAGEN, Inc., Germantown, MD, USA; accessed 1 March 2024).

## 5. Conclusions

As fentanyl abuse progresses further into a pandemic, the rate of overdose fatalities and survivors will continue to expand. Little is known about the long-term organ impacts after overdose survival in humans. While no overt cardiopulmonary phenotype has been reported yet, the third wave of the opioid epidemic (beginning around 2013 and focused on fentanyl) is still in its infancy, with unknown future health implications associated with overdose survival. In this study, we investigated the ability of fentanyl overdose from a single bolus to elicit changes in a panel of cytokines, chemokines, and growth factors in the cardiopulmonary system at 40 min, 6 h, 24 h, and 7 d post-exposure. Generally, the analytes were decreased in the heart and lungs, increased in plasma, and unchanged in the cerebral cortex. The heart was heavily impacted across all time points. LD50 doses yielded a distinct response in contrast to HNLD and LD10 doses, which may be partially attributable to survivor bias. Across all samples, proteins with the greatest contribution to variance, as a function of largest-magnitude changes in concentration, were eotaxin-1, IL-33, and BTC. These and many other proteins were generally decreased in the hearts and lungs, yet they were increased or unchanged in plasma, suggesting resistant, persistent anti-inflammatory environments that may impact tissue remodeling and infection risk. Given the disparities between plasma and heart/lung concentrations of biomarkers in this study, confirmatory studies in humans may be hindered by sample availability, and surrogate markers of cardiac changes (e.g., ECG or MRI) have not been explored in vulnerable individuals. Nonetheless, the results of this study reveal nonclassical immunomodulatory effects of fentanyl that warrant further elucidation, for the endurance of the expanding population of overdose survivors.

## Figures and Tables

**Table 1:** Analytes showing maximum contribution (>|0.2|) to PC1 and PC2 of PCA plot depicted in Figure S3.

Analyte	PC1 Loading		Analyte	PC2 Loading
Eotaxin-1	−0.5097		BTC	−0.2070
BTC	−0.3312		IL-7	−0.2053
IL-7	−0.3155		MIP2α	0.2133
IL-3	−0.3146		ENA78	0.2542
IL-18	−0.2799		IL-25	0.2668
BAFF	−0.2647		BAFF	0.2696
MIP-1α	−0.2055		RANTES	0.2858
GRO-α	−0.2053		IL-33	0.4629

## Data Availability

The data presented in this study are available on request from the corresponding author due to concerns about AI data scraping.

## Supplementary Materials

The following supporting information can be downloaded at: https://www.mdpi.com/article/10.3390/ph17070941/s1, Figure S1: Spatiotemporal distribution of analytes in cardiopulmonary system after fentanyl overdose; Figure S2: Two-dimensional principal component analysis (PCA) of individual tissues; Figure S3: Two-dimensional principal component analysis (PCA) of all samples; Figure S4: Individual loading of first five principal components.
