# Supplementary material for: Fentanyl Overdose Causes Prolonged Cardiopulmonary Dysregulation in Male SKH1 Mice
Source: Pharmaceuticals (Basel). 2024 Jul 14;17(7):941. doi: 10.3390/ph17070941 (PMC11279777; doi:10.3390/ph17070941)

IL-25	IL-9	RANTES	MIP-2α	ENA78	IL-18	BAFF	IL-33

Cortex

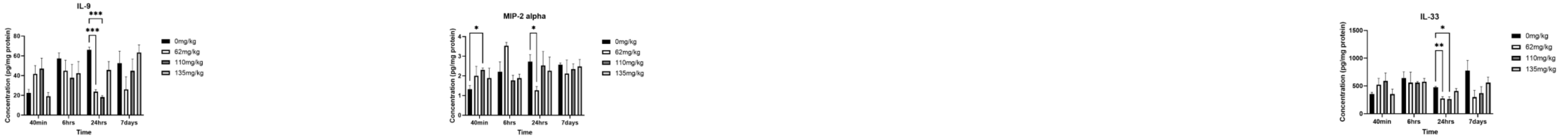

Lungs

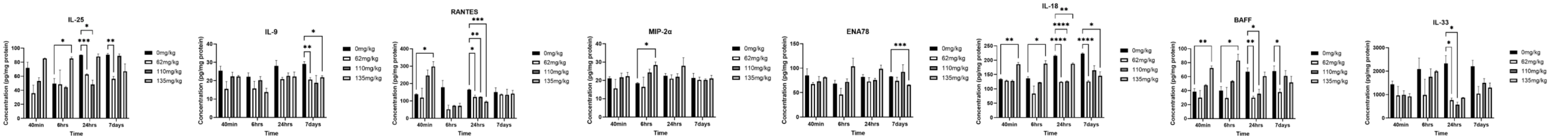

Heart

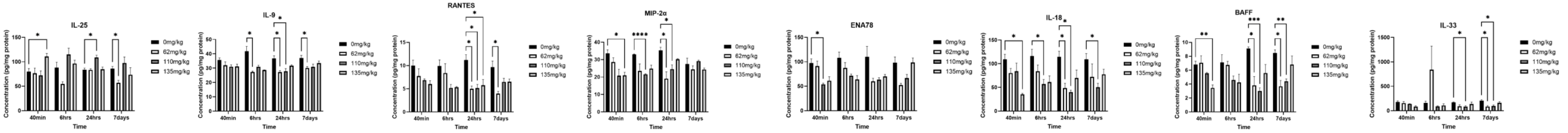

Plasma

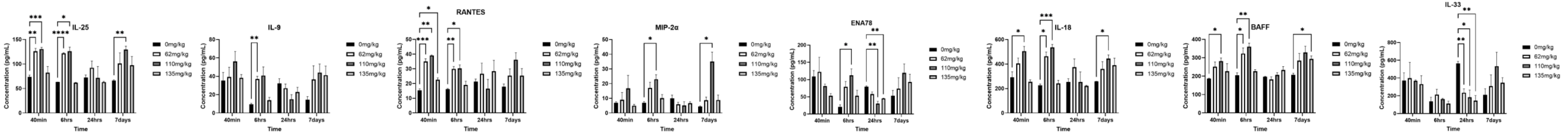

IP-10	IL-13	IL-23	GRO-α	MIP-1α	MCP-3	IL-7Rα	BTC

Cortex

Lungs

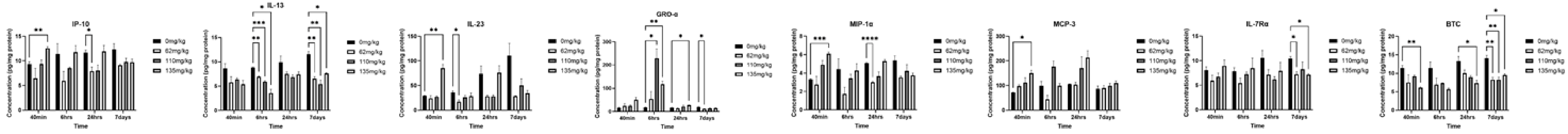

Heart

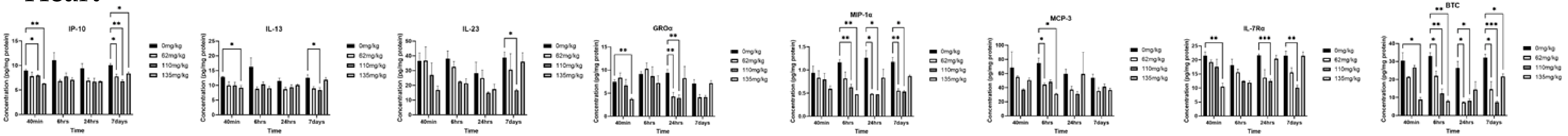

Plasma

IL-2	IL-4	TNF-α	IL-1α	Eotaxin-1	IL-6	IL-17α	RANKL

Cortex

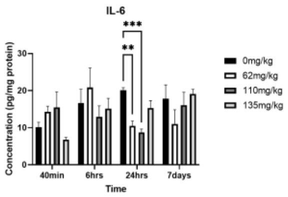

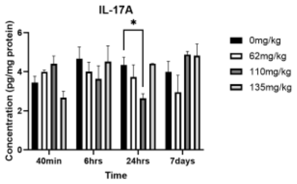

Lungs

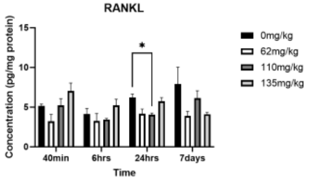

Heart

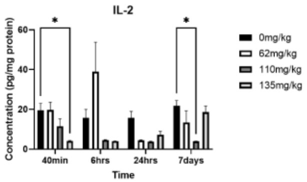

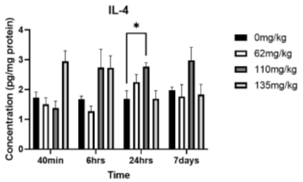

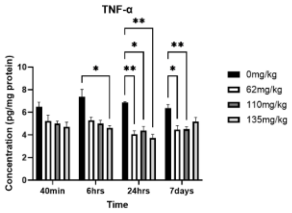

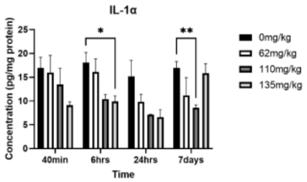

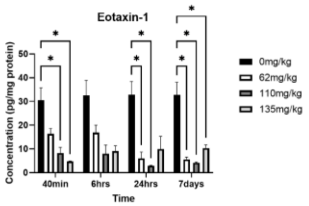

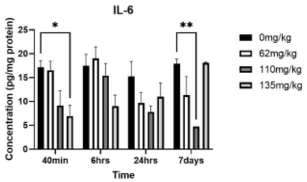

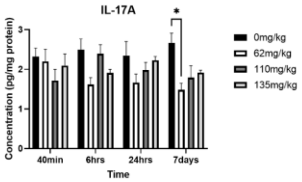

Plasma

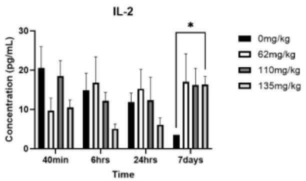

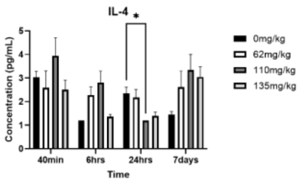

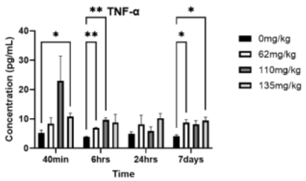

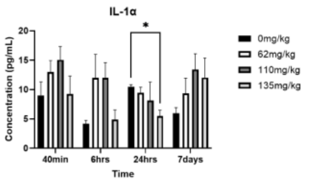

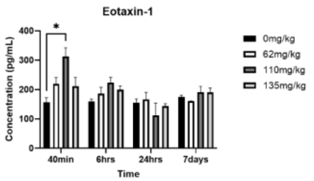

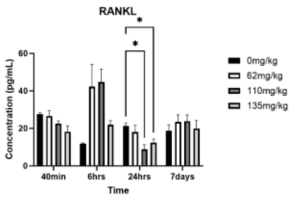

Lungs

LIF	VEGF-A	MCP-1	IL-19

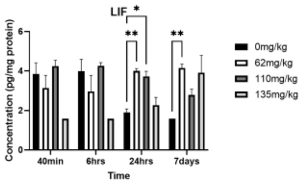

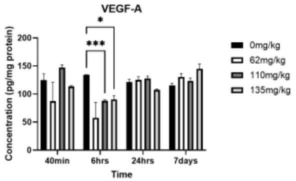

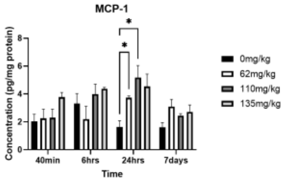

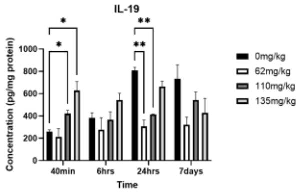

Heart

IL-3	M-CSF	IL-27	IL-28	IL-7

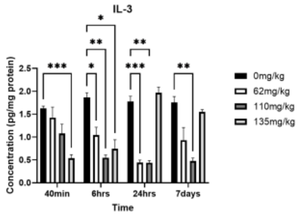

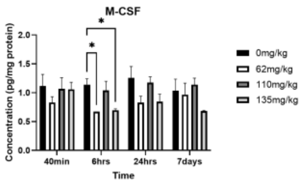

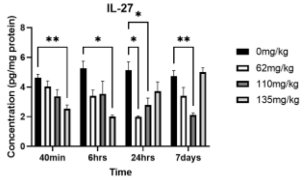

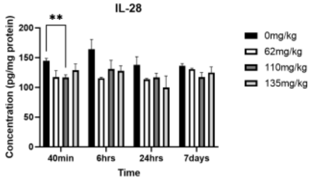

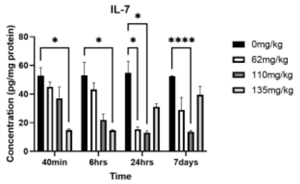

Plasma

IL-15	IL-2R	IL-31

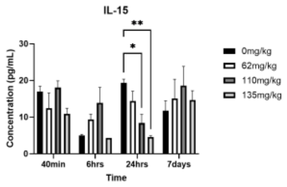

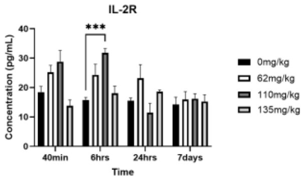

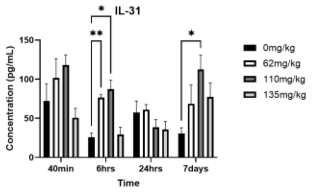

Supplement: Supplementary file 1 [file pharmaceuticals-17-00941-s001.zip › figS1.pdf]
