# Supplementary figures and images for: Fentanyl Overdose Causes Prolonged Cardiopulmonary Dysregulation in Male SKH1 Mice
Source: Pharmaceuticals (Basel). 2024 Jul 14;17(7):941. doi: 10.3390/ph17070941 (PMC11279777; doi:10.3390/ph17070941)

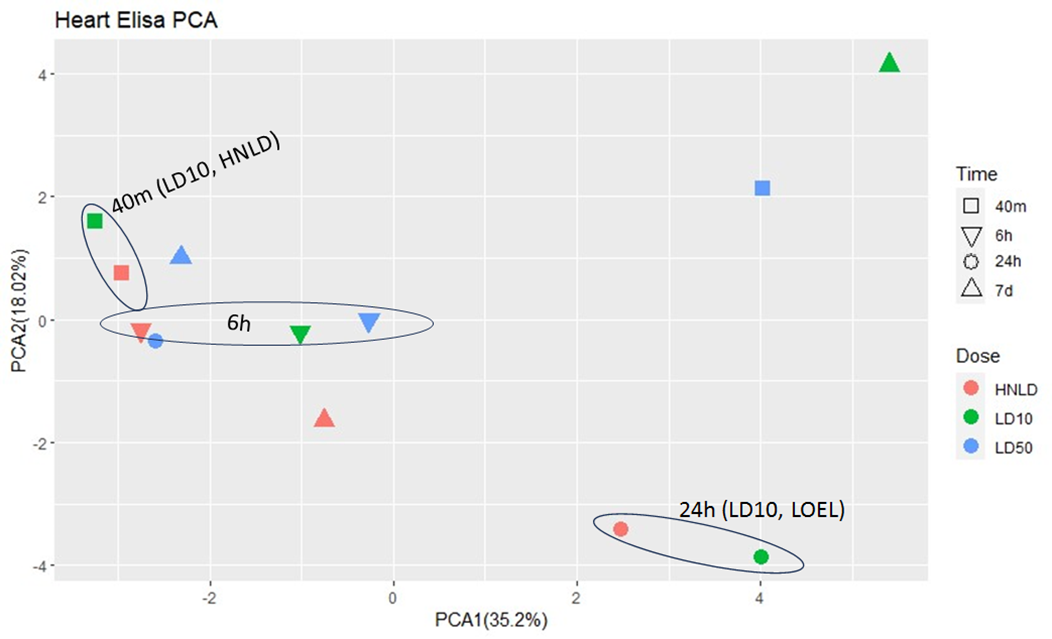

Supplement: Supplementary file 1 [file pharmaceuticals-17-00941-s001.zip › figS2a.tif]

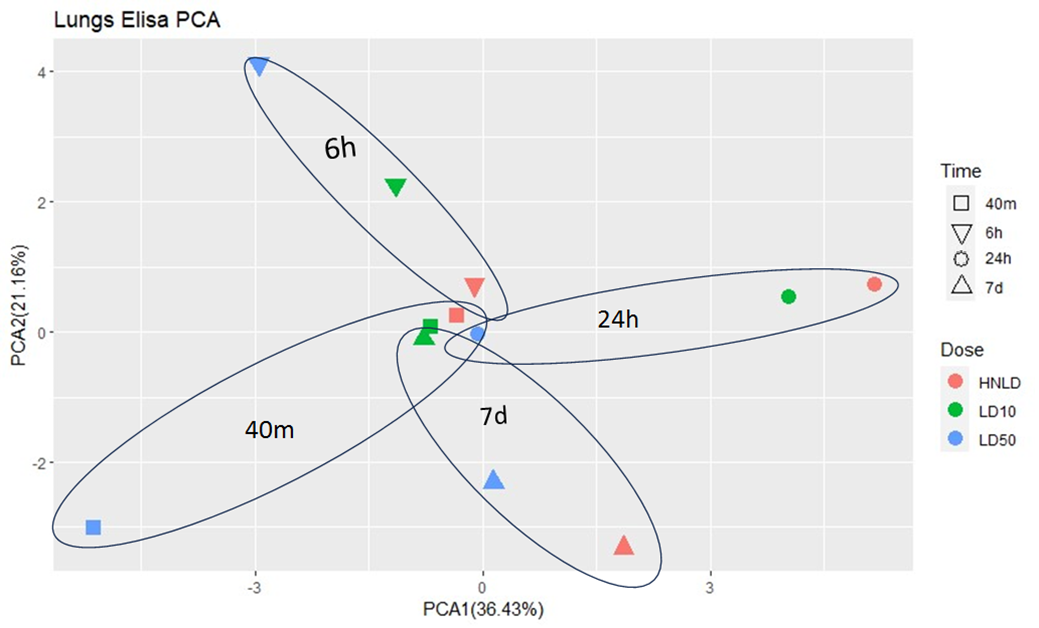

Supplement: Supplementary file 1 [file pharmaceuticals-17-00941-s001.zip › figS2b.tif]

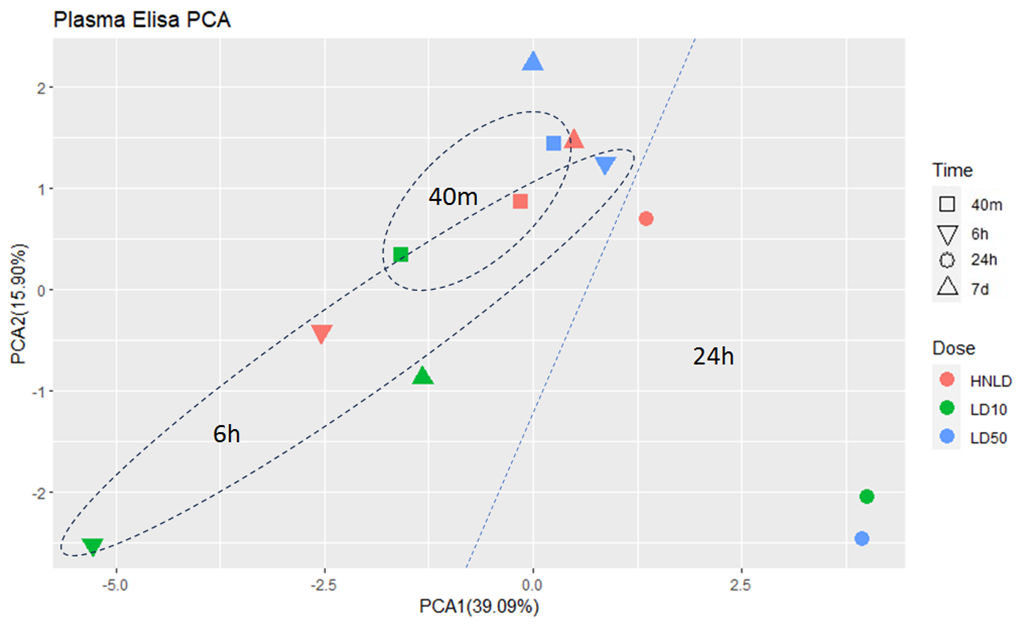

Supplement: Supplementary file 1 [file pharmaceuticals-17-00941-s001.zip › figS2c.tif]

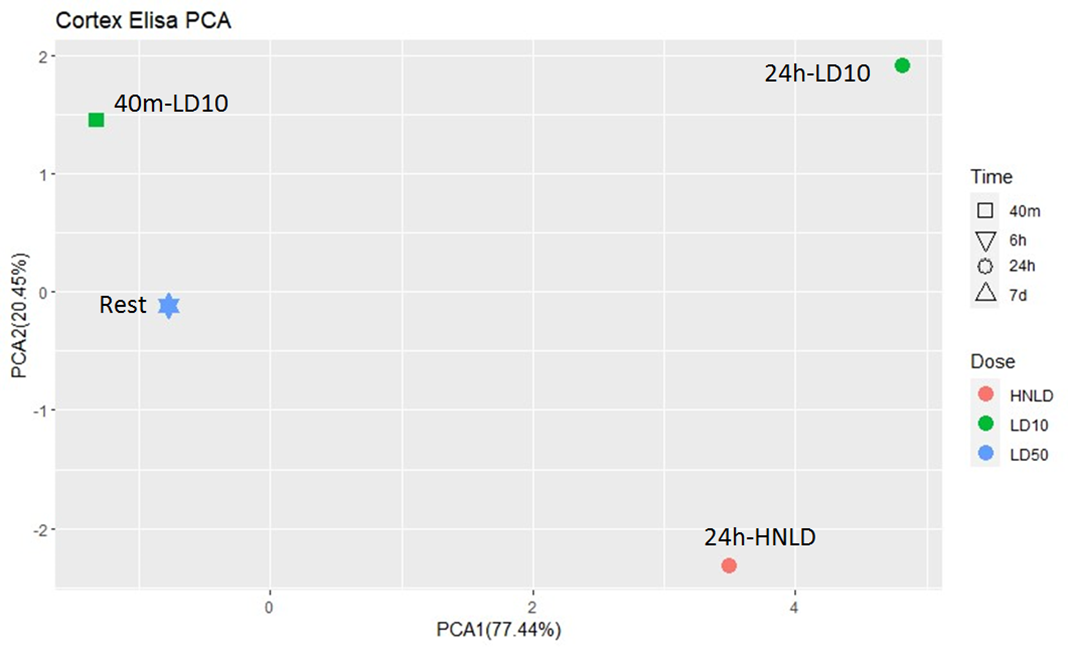

Supplement: Supplementary file 1 [file pharmaceuticals-17-00941-s001.zip › figS2d.tif]

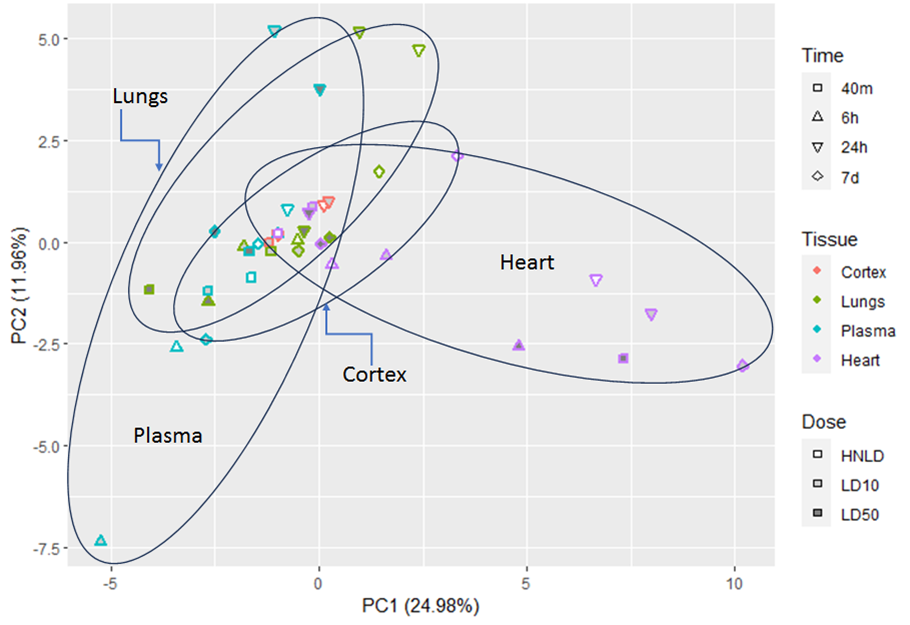

Supplement: Supplementary file 1 [file pharmaceuticals-17-00941-s001.zip › figS3.tif]

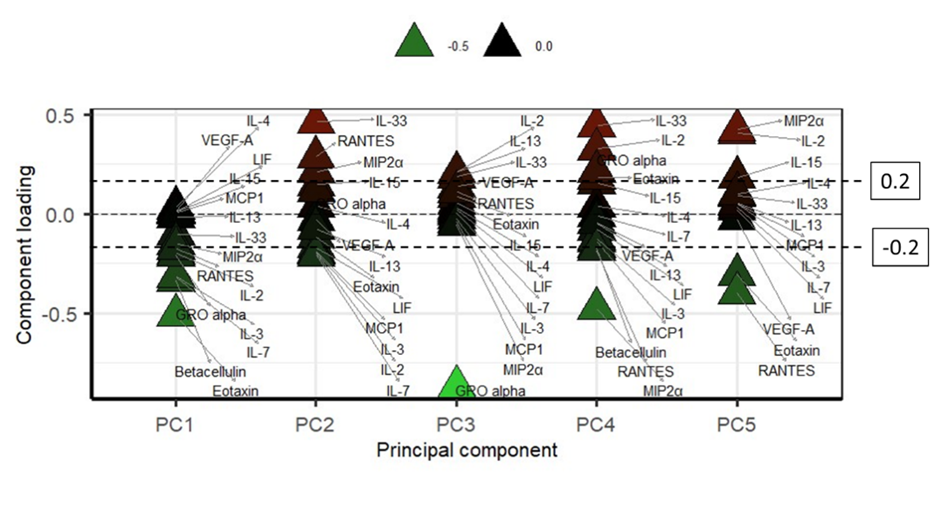

Supplement: Supplementary file 1 [file pharmaceuticals-17-00941-s001.zip › figS4.tif]
